# Cranberry: A Promising Natural Product for Animal Health and Performance

**DOI:** 10.3390/cimb47020080

**Published:** 2025-01-27

**Authors:** Sahdeo Prasad, Bhaumik Patel, Prafulla Kumar, Pranabendu Mitra, Rajiv Lall

**Affiliations:** 1 R&D LifeSciences LLC, 8801 Enterprise Blvd, Largo, FL 33773, USA; 2 Department of Immunotherapeutics and Biotechnology, Texas Tech University Health Science Center, Abilene, TX 79601, USA; 3 Department of Kinesiology, Health, Food & Nutritional Sciences, University of Wisconsin-Stout, Menomonie, WI 54751, USA

**Keywords:** cranberry, antimicrobial, antioxidant, immunomodulatory, gut health, livestock animals

## Abstract

Cranberries are a distinctive source of bioactive compounds, containing polyphenols such as flavonoids, anthocyanins, phenolic acids, and triterpenoids. Cranberries are often associated with potential health benefits for the urinary tract and digestive system due to their high antioxidant, anti-inflammatory, antimicrobial, and immunomodulatory properties. Cranberry induces the production of antioxidant enzymes, suppresses lipid peroxidation, reduces inflammatory cytokines, modulates immune cells, maintains gut microbiota, and inhibits bacterial adhesion and growth. Cranberry polyphenols also have metal-binding motifs that bind with metals, particularly zinc and iron. The combination of cranberry polyphenols and metals displays increased biological activity. In this review, an attempt is made to describe the physiological properties and health benefits of cranberries for livestock, including poultry, swine, canine, feline, and ruminant animals, as either feed/food or as supplements. Cranberry, and/or its components, has the capability to potentially control infectious diseases like diarrhea, urinary tract infection, gut integrity, and intestinal probiotic health. Moreover, cranberries show efficacy in suppressing the growth of pathogenic microorganisms such as *Salmonella* species, *Campylobacter* species, *Streptococcus* species, and *Enterococcus* species bacteria. Thus, cranberry could be considered as a potential natural feed additive or food supplement for animal health improvement.

## 1. Introduction

Cranberry is an evergreen dwarf shrub or trailing vine that is about 7 feet in height and has a creeping habit. It is commercially cultivated in the United States and Canada. In North America, cranberries grow on trailing vines in bogs and marshes and produce ‘large’ cranberries, which are referred to as Vaccinium macrocarpon [1]. The ‘small’ cranberry, also called *Vaccinium oxycoccus,* is found in marshy land and cultivated in northern North America and Asia and in northern and central Europe. In Britain, cranberry may refer to the native species *Vaccinium oxycoccos* [2].

The name cranberry derives from the Middle Low German kraanbere (English translation, cranberry). It has been postulated that the missionary John Eliot named cranberry in 1647 for the first time in English. According to the Food and Agriculture Organization of the United Nations, the world production of cranberries has increased dramatically from 260,480 tons in 1994 to 582,924 tons in 2022. The U.S. is the primary producer (365,500 tons), followed by Canada (209,205 tons) and Turkiye (4305 tons) (UNFAO). Cranberry is mostly produced in Wisconsin and this area produced 59 percent of the U.S. crop in 2021. Other leading cranberry-producing states include Massachusetts, New Jersey, and Oregon (USDA NASS 2022). Since cranberries are only produced in a few countries around the world, the amount currently available will probably not be enough if demand increases. Also, the price of cranberries is very high. Worldwide, the average unit price paid by importers for cranberries was $4133 per ton in 2023 (www.worldstopexports.com, accessed on 22 January 2025). Cranberry has nutritional value and contains several polyphenols, which hold its potential for providing health benefits.

## 2. Composition of Cranberry

Cranberry fruits represent a rich source of bioactive compounds. Although raw cranberries are composed of 87% water and 12% carbohydrates and contain negligible proteins and fats, they are rich in dietary fiber, vitamins, and minerals, including magnesium, calcium, potassium, vitamin C, and vitamin E [3]. Moreover, cranberries are considered among the important dietary sources of bioactive compounds, like polyphenols and terpenes.

Cranberries have a wide range of both water-soluble and fat-soluble vitamins. The content of vitamins varies with the cultivar. The highest concentration of vitamin C was identified in the “Pilgrim” (20.74 mg/100 g fresh matter) cultivar, and the lowest content was found in the “Red Star” (10.07 mg/100 g fresh matter) cultivar [4]. The amount of vitamin E does not vary significantly, and it ranges from 1.31 mg/100 g to 1.56 mg/100 g (fresh matter) in small cranberries from of natural habitats. Small amounts of vitamin B1 and B2 have also been detected in both small and large cranberries. Cranberries accumulate an average of 0.32 mg/100 g vitamin B1 and 0.23 mg/100 g vitamin B2. Minerals are also an important component of cranberries. In cranberry, total minerals account for 0.19–0.28% of fresh weight and 1.08–2.45% of dry weight [5]. Cranberries also comprise fibers. The content of soluble pectin fiber ranges from 0.75% in “RedStar” cultivar to 1.11% in “Franklin” cultivar [4].

### 2.1. Polyphenols

**Figure 1:**
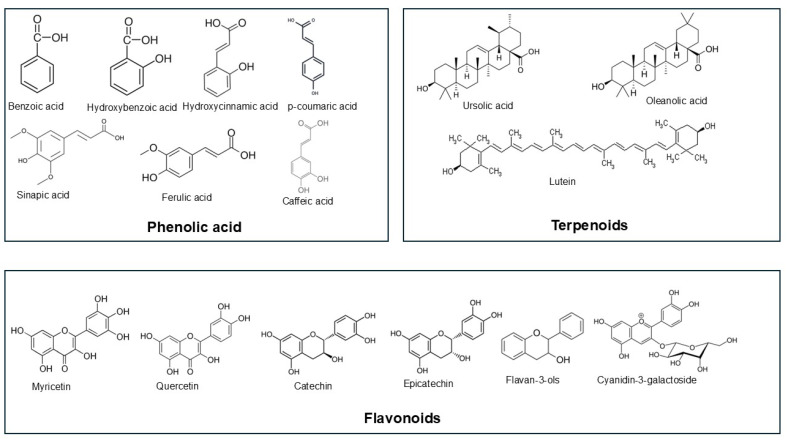
The chemical structure of major polyphenols present in cranberry fruit.

The polyphenols present in cranberries include phenolic acids, flavonoids, anthocyanins, and tannins [3]. Figure 1 shows the major polyphenols present in cranberry fruit. The numbers and amounts of polyphenols in cranberry vary with the stage of ripening. Immature cranberries contain the lowest amounts of polyphenols, and this level increases with the ripening of fruit. Cranberry fruit contains the highest amounts of flavan-3-ols (41.5–52.2%), followed by flavonols (18.6–30.5%), anthocyanins (8.0–24.4%), and then phenolic acids (5.0–12.1%) [6]. Cranberry also contains several organic acids, such as quinic, citric, malic, and benzoic acids. These bioactive compounds have the power to positively affect the health of animals [7].

#### 2.1.1. Phenolic Acids

Cranberry contains a large number of phenolic acids, which include benzoic acid and hydroxybenzoic acid as well as hydroxycinnamic acids. Hydroxycinnamic acids constitute p-coumaric, caffeic, sinapic, and ferulic acids [8]. The quantity of phenolic acids in ripened cranberries varies with the different cultivars. However, total phenolic acids ranged from 327 mg to 649 mg per 100 g dry matter [4].

#### 2.1.2. Flavonoids

Flavonoids are important polyphenols in plants as they have multiple biological activities. The level of flavonoids in cranberries normally ranges from 860 mg to 1283 mg per 100 g dry matter [4]. However, these contents vary depending on the cultivars. Several flavonoids have been identified in cranberry, including myricetin, quercetin, catechin, epicatechin, proanthocyanidins, and flavan-3-ols. Anthocyanins, which are a subgroup of flavonoids, are among the major polyphenols present in cranberry fruits. The commonly recognized anthocyanins are cyanidin-3-galactoside, cyanidin-3-arabinoise, cyanidin-3-glucoside, peonidin-3-galactoside, peonidin-3-arabinoside, and peonidin-3-glucoside [9]. Cranberry fruit proanthocyanidins, which are members of the flavonoid family, have unique molecular structures. The bioactivity and bioavailability of cranberries have been shown to be influenced by these structural features of the proanthocyanidins [10].

### 2.2. Terpenoids

The flavor and aroma of cranberries are associated with the presence of terpenes that include volatile compounds. Depending on the number of isoprene units, terpenoids are classified as mono (2 isoprene units), sesqui (3 units), di (4 units), tri (6 units), and so forth [11]. Cranberry fruits mostly contain the triterpenoids ursolic acid and its isomer, oleanic or oleanolic acid. The content of triterpenoids ranges from 252 to 320 mg per 100 g of dry matter of cranberry [6]. Ursolic acid alone is reported to be present at a rate of 46–109 mg per 100 g of whole cranberry fresh fruit from different cultivars [12]. However, these contents may vary with the maturing stage of fruits and cranberry cultivars. In addition, these cranberries contain carotenoid lutein, as well as other carotenoids in lesser quantities.

## 3. Physiological Properties of Cranberry

**Figure 2:**
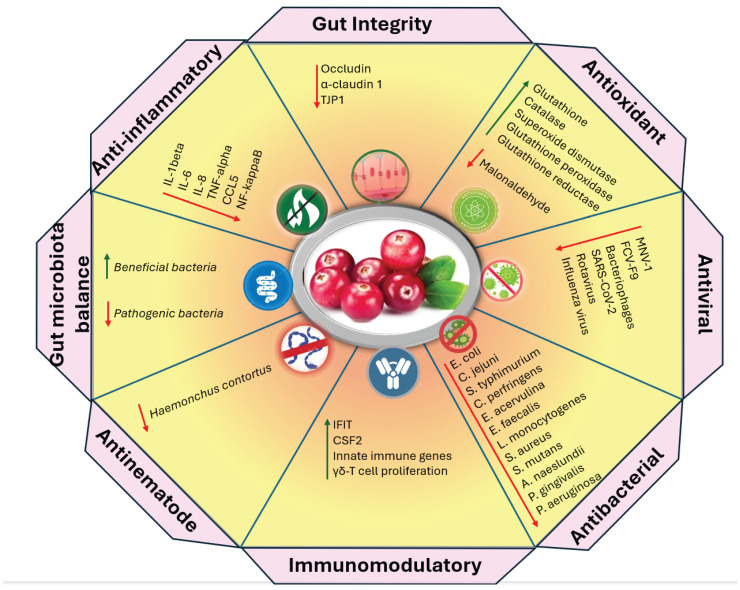
Antioxidant, anti-inflammatory, antibacterial, antiviral, antinematode, immunomodulatory, gut-microbiota-balancing, and gut-integrity-supporting properties of cranberry, both in vitro and in animals. 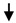 Downregulation, 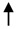 Upregulation.

Cranberry is not only known for its nutritional value, but also because of its various health-promoting characteristics. Accumulated evidence describes how cranberry exhibits antioxidant [4], anti-inflammatory [13], antibacterial [14], antiviral [15], anti-diabetic, anti-obesity [16], anticancer [17], cardioprotective [18], neuroprotective [19], immunomodulatory [20], and digestion-promoting effects [21] (Table 1). The physiological properties of cranberries, as shown in Figure 2 (derived from Table 1), are associated with bioactive components, including various polyphenols and terpenoids.

### 3.1. Antioxidant Activity

Antioxidants are substances that neutralize the free radicals produced by environmental pollutants and unhealthy food, as well as during metabolism in the body, and thus protect cells from free radical-induced damage. If free radicals are not destroyed, their presence can contribute to the initiation of various diseases like cardiovascular diseases, diabetes, aging, inflammatory bowel disease, cancer, etc. [57]. Cranberry fruit is known for its richness in antioxidants, as it contains various polyphenols and terpenoids [4]. Antioxidant molecules of cranberry scavenge free radicals and neutralize reactive oxygen species (ROS) that oxidize biological matter and cause oxidative stress. Using the DPPH and ABTS methods, Balawejder et al. [22] showed that highbush cranberry extract has high antioxidant activity (2271.2 mg TE per100 g dry matter and 2271.2 mg Trolox Equivalents (TE) per 100 g dry matter respectively). Recently, the antioxidant activity of cranberry was determined using an in vitro method. Cranberry exhibited a DPPH antioxidant capacity of 6.44 mg TE/g dry weight, an ABTS antioxidant capacity of 12.30 mg TE/g dry weight, and a FRAP value of 24.39 mg FeSO_4_·7H_2_O/g dry weight [23]. Further, studies performed on the *Saccharomyces cerevisiae* model showed a significant decrease in cellular ROS level, confirming its strong antioxidative nature [22].

As polyphenolic contents vary by cultivar, Urbstaite et al. [58] compared the antioxidant capacity of “Woolman” and “Le Munyon” cultivars. They determined that the greatest radical scavenging activity was seen in the “Woolman” (849.75 ± 10.88 µmol TE/g) fruit sample and the highest reducing activity was seen in the “Le Munyon” (528.05 ± 12.16 µmol TE/g) fruit sample [58]. Furthermore, the cultivars “Franklin”, “Howes”, and “Stevens” showed higher antioxidant activity (ABTS: 226, 264, 246; FRAP: 102, 139, 124; DPPH: 235, 320, 284 μmolTE/g dm) than “Ben Lear”, “Pilgrim”, and “Red Star”, as these cultivars were characterized by the highest concentrations of total polyphenols and triterpenoids [4]. Thus, a correlation is seen whereby the total polyphenol content has a link with the antioxidant and reductive activity of the cranberry extracts.

In a hypercholesterolemic rat model, dietary cranberry powder was found to restore lipopolysaccharide (LPS)-induced diminished antioxidant levels [24]. Cranberry juice was also found to improve antioxidant status in orchidectomized (one or both testicles are removed) rats. The ingestion of 27% and 45% cranberry juice for 120 days by the rats overcame oxidative stress by increasing plasma antioxidant capacity and superoxide dismutase activity and decreasing nitrate and malondialdehyde concentrations, indicating its protective effect against oxidative damage [25]. Cranberry extract (100 mg/kg/day) alleviated the doxorubicin-induced suppression of catalase, superoxide dismutase, glutathione, glutathione peroxidase, and glutathione reductase antioxidant enzymes in rats. This extract also mitigated the increased levels of malondialdehyde and protein carbonyls in the cardiac tissues of rats [59]. It has also been observed that cranberries induce antioxidant enzymes in broilers. The supplementation of 0.5 or 1.0% cranberry pomace in broilers resulted in increased levels of Nrf, Gpx2, and HO-1 antioxidant enzymes [26]. Thus, cranberry induced antioxidative effects by enhancing antioxidant enzymes and inhibiting the production of free radicals in both in vitro and in vivo.

### 3.2. Anti-Inflammatory Activity

The phrase anti-inflammatory refers to a substance that reduces inflammation by blocking substances in the body that cause inflammation. It is the body’s response to harmful stimuli, such as pathogens, damaged cells, or irritants. Although short-term (acute) inflammation is beneficial to the body as it protects against infectious microorganisms, chronic inflammation causes various diseases including cancer, heart disease, aging, etc. [60].

Cranberries exert anti-inflammatory effects in both in vitro and in animal models. Studies showed that cranberry concentrate reduces LPS-induced inflammation in human gingival fibroblasts, osteosarcoma-derived osteoblasts, and activated macrophages. In LPS-stimulated macrophages, cranberry concentrates (50 and 100 µg/mL) downregulated pro-inflammatory cytokine expression IL-6 and IL-8, and upregulated anti-inflammatory IL-10 expression [13]. Another study further confirmed that cranberry potently inhibits LPS-induced pro-inflammatory cytokine and chemokine responses. The treatment of the cranberry fraction prior to stimulation by LPS resulted in the suppression of IL-1beta, IL-6, IL-8, TNF-α, and RANTES (Regulated on Activation of Normal T-cell Expressed and Secreted) production in macrophages [27].

Cranberry proanthocyanidins were also shown to alter *P. gingivalis*-induced inflammatory responses in human oral epithelial cells. Cranberry proanthocyanidins inhibited the secretion of IL-8 and chemokine (C-C motif) ligand 5 (CCL5) in epithelial cells after their release was increased by *P. gingivalis*. The anti-inflammatory effect of cranberry proanthocyanidins was found to be associated with the suppression of the nuclear factor–kappaB (NF-kappaB) p65 pathway [28]. In THP-1 cells, cranberry extracts decreased the TNF-*α* expression induced by LPS, further indicating their anti-inflammatory properties [29]. Taken together, these data indicate that cranberry has anti-inflammatory effects and may protect cells from inflammatory damage.

### 3.3. Antimicrobial Activity

Cranberry is a potent antimicrobial agent against various pathogenic and food-borne microorganisms. Cranberry extracts are reported to suppress the growth of various types of pathogenic bacteria, including both Gram-negative (*Escherichia coli* and *Salmonella typhimurium*) and Gram-positive (*Enterococcus faecalis, Staphylococcus aureus,* and *Listeria monocytogenes*) bacteria [14]. In another study, the antimicrobial action of cranberry juice against oral pathogens was assessed, and it was found that cranberry juice (0.5 mL/mL) expressed a bactericidal effect on the growth of *S. mutans* and *Actinomyces naeslundii* [30]. It was found to be more potent than other berries, like blueberry acai berry, raspberry, and strawberry, in inhibiting the growth of *S. aureus* bacteria. Furthermore, it was observed that the antimicrobial effect of cranberry juice was not associated with the acidity of the berries as neutralized juices were almost as effective [31].

Cranberry juice concentrates have also shown antimicrobial activities against the panel of microorganisms like *Streptococcus mutans*, *Aggregatibacter actinomycetemcomitans*, *Enterococcus faecalis*, *Porphyromonas gingivalis*, and *Tannerella forsythia*, which are responsible for periapical and periodontal infections. Cranberry juice concentrates showed a minimum inhibitory concentration (MIC) value of 50 mg/mL, while the minimum bactericidal concentration (MBC) value was 100 mg/mL. They also exhibited antiadhesion (83–90%) and antibiofilm activity (70–85%) and thus inhibited bacterial cell attachment and induced susceptibility to antibiotics and the host immunological system (HR 2017). A study conducted by Ulrey et al. [32] confirmed that proanthocyanidins from cranberries disrupt biofilm formation and further kill *Pseudomonas aeruginosa*. Proanthocyanidins suppressed the swarming motility of *P. aeruginosa* and potentiated the antibiotic activity of gentamicin in the *Galleria mellonella* in vivo model of infection [32]. In another in vivo model of *Drosophila melanogaster*, cranberry proanthocyanidins demonstrated anti-virulence activity against *Pseudomonas aeruginosa*. Proanthocyanidins reduced the production of virulence determinants and protected *D. melanogaster* from fatal infection with *P. aeruginosa* PA14 [33].

Cranberry not only kills pathogenic Enterobacteriaceae bacteria but is also found to increase the abundance of Bacteroidaceae microbiota in the gut. It was found that the cranberry component, salicylate, exerted antimicrobial activity against *E. coli* and elevated probiotics [34]. In a broiler chicken study, supplementation of 1 or 2% of cranberry pomace into a basal diet suppressed undesirable bacteria (*Synergistaceae* and *Desulfovibrio*, and *Fusobacteriaceae*) and increased beneficial bacterial taxa (including *Bifidobacterium*, unclassified_*Rikenellaceae*, and *Faecalibacterium*) in the intestine [39]. This finding indicates the antipathogenic and gut-supportive properties of cranberry. Cranberry also showed antimicrobial activity by suppressing urinary tract infection (UTI) development in dogs. The supplementation of cranberry to the dogs with a history of recurrent UTIs did not lead to occurrence of UTIs after 6 months [40], indicating its strong antimicrobial properties in livestock like dogs.

The cranberry extract was tested in patients with a single urinary tract infection (UTI), and it was found that patients’ complaints decreased from day 3 of treatment and their well-being increased with the use of cranberry. Interestingly, on day 7, the well-being efficacy of cranberry in patients was higher than that of the antibiotic fosfomycin. Thus, it has been suggested that cranberry can serve as an alternative to antibiotics for simple UTIs [35]. In another clinical study on 145 women, the treatment of cranberry proanthocyanidins (2 × 18.5 mg daily for 24 weeks) showed a preventive impact on symptomatic UTI recurrence in women who experienced less than 5 infections per year. This high dose of cranberry did not show major side effects for the patients [36].

The use of cranberry extract in combination with strains of Lactobacilli was found to be effective for preventing recurrent UTIs in pre-menopausal adult women. The supplementation of this combination twice daily for 26 weeks significantly improved the recurrence of UTIs compared to placebo [37]. Another combination of cranberry with propolis was found to reduce the frequency of cystitis in women with recurrent acute cystitis. In a multicenter, placebo-controlled, randomized study of women aged >18 years with at least 4 episodes of cystitis, cranberry and propolis supplementation significantly reduced the incidence of UTIs during the first 3 months and delayed the onset of an episode of cystitis [38].

### 3.4. Antiviral Activity

In addition to antibacterial activity, cranberry extracts, including proanthocyanidins, are effective against many viruses, including foodborne viral surrogates, murine norovirus (MNV-1), feline calicivirus (FCV-F9), ϕX-174 (ssDNA) bacteriophage, MS2 (ssRNA) bacteriophage, and phiX-174 (ssDNA) bacteriophage. Cranberry juice and cranberry proanthocyanidins were shown to reduce the infectivity of viruses within 1 h at room temperature when viruses at titers of 5 log10 PFU/mL were mixed with equal volumes of cranberry juice at pH 2.6 [15]. In another study, non-cytotoxic concentrations of cranberry pomace extract, a byproduct obtained during cranberry juice extraction, blocked dengue virus and zika virus infection in human Huh7.5 and A549 cell lines, respectively. Cranberry pomace extract prevented the attachment of the virus to the cell surface by directly acting on viral particles, thus inhibiting the entry of the virus into the host cell [41]. Cranberry extract was also found to be effective against the Hazara virus, which is a tick-borne arbovirus. It has been shown that cranberry targets the viral replication cycle at its early stages, like adsorption to target cells. Furthermore, cranberry extract directly interacts with Hazara virus particles and subsequently impairs the attachment of viruses to cell surface receptors and thus exerts virucidal effects [42].

Recently, cranberry phytochemicals have been shown to possess antiviral activities against SARS-CoV-2. These are exerted by targeting its main protease (M^pro^) enzyme. Anthocyanins extracted from frozen cranberry exhibited potent protease enzyme inhibitory activity, with an IC_50_ of 23.58 μg/mL, while cyanidin 3-O-galactoside, a class of anthocyanin, exhibited enzyme inhibitory activity with an IC_50_ of 9.98 μM. This finding indicates that cranberry can be used for therapeutic interventions against SARS-CoV-2 [43]. Cranberry polyphenols inhibit viral infectivity via modifications in cellular physiological events and physical factors. In a cell-free viral suspension, cranberry proanthocyanidins induced the aggregation of rotavirus and caused the destruction of rotavirus capsid protein VP6. Cranberry also had a significant protective effect on the epithelial cell tight junction (TJ), as observed by alterations in transepithelial electrical resistance (TEER) and by a decrease in the signal intensity of the TJ α-claudin 1 protein [44]. Moreover, cranberry proanthocyanidins with the epigallocatechin gallate (EGCG) of green tea showed antiviral synergy against rotavirus. EGCG (30 µg/mL) and proanthocyanidins (25 µg/mL) alone reduced rotavirus titers by 3 and 13%, respectively. However, rotavirus titers were reduced by 32% when both flavonoids were used in combination, indicating that their combination has synergistic antiviral effects [45].

As cranberry extract inhibits the adhesion of bacteria, testing was performed to determine whether cranberry can prevent viral attachment to target cells. It was found that the cranberry extract inhibited the replication of *influenza A* virus and *B* virus (IAV, IBV) in vitro. Cranberry polyphenols prevented the attachment and entry of IAV and IBV into target cells and exerted virucidal activity. Influenza viral particles lost their infectivity, which was probably due to the interaction of cranberry polyphenols with the ectodomain of viral hemagglutinin (HA) glycoprotein [46]. Because of its antiadhesive properties, cranberry also has been shown to prevent the adsorption of HSV-1 and HSV-2 to target cells. Cranberry juice and its proanthocyanidins target the viral envelope glycoproteins gD and gB, thus resulting in a loss of infectivity of HSV particles [47]. The high-molecular-weight nondialyzable materials (NDM) of cranberry juice were found to be more effective in inhibiting influenza virus adhesion to cells and its infectivity. Because influenza virus surface glycoproteins, hemagglutinin and neuraminidase, are involved in viral replication and in the infection process, NDM inhibited influenza virus-induced hemagglutination, thereby suppressing viral replication at a concentration of 125 micron/mL or lower, which was at least 20-fold lower than that of cranberry juice [48]. In addition, NDM inhibits the neuraminidase enzymatic activity of influenza A and B strains, as well as that of Streptococcus pneumoniae [49]. Thus, cranberry exerts antiviral activity by targeting viral proteins.

### 3.5. Immunomodulatory Effect

Cranberry proanthocyanidins play an immunity-promoting role in organisms. In the *Caenorhabditis elegans* model, proanthocyanidins have been found to increase host innate immunity against *Vibrio cholerae* infection. Proanthocyanidins increased the expression of *C. elegans* innate immune genes, such as clec-46, pqn-5, clec-71, fmo-2, and C23G10.1, which helped to fight against *V. cholerae* infection [20]. Recently, cranberry proanthocyanidins have been shown to reverse the effects of reflux-induced bacterial, inflammatory, and immune-implicated proteins and genes in rats, including Ccl4, Cd14, Crp, Cxcl1, Il6, Il1b, Lbp, Lcn2, Myd88, Nfkb1, Tlr2, and Tlr4 [50]. These are helpful in ameliorating reflux-induced dysbiosis, inflammation, and cellular damage. Additionally, cranberry extracts exhibited a response to the LPS in the human monocytic cell line THP-1. Several immune-related genes were found to be responsive to cranberry extracts, including interferon-induced protein with tetratricopeptide repeats 1 and 3 (IFIT 1 and 3), macrophage scavenger receptor 1 (MSR1), and colony-stimulating factor 2 (CSF2), indicate that cranberry polyphenols have protective effects through modulation of immune systems [29].

Cranberry phytochemicals also increase immunity by upregulating γδ-T cell proliferation. γδ-T cells are located in the epithelium and serve as a first line of defense. In a human clinical study, the daily consumption of a cranberry beverage (450 mL daily) caused an almost five times higher proliferation index of γδ-T cells after 10 weeks. Increased levels of γδ-T cells are further found to be associated with a reduced number of symptoms linked with a cold and flu [51]. Additionally, cranberry proanthocyanidin also decreased pulmonary immune responses, such as keratinocyte-derived cytokine and polymorphonuclear cell recruitment in bronchoalveolar lavage fluid in a pneumonia mouse model with *E. coli*. Moreover, the use of cranberry reduced mortality by more than half in mice inoculated with *E. coli* [61]. In a broiler model, the supplementation of cranberry NDM (2 and 4 mg/mL/bird) increased the serum IgM level (*p* < 0.05) and the antibody titers against IBDV, indicating its immune-stimulating effects in chickens [52].

### 3.6. Cranberry in Gut Microbiota

The gut microbiome plays a critical role in the etiology of various diseases through complex interactions with the host’s immune, metabolic, and physiological systems. Cranberry has been found to be beneficial for maintaining the gut microbiota in animals, mainly through altering physiological systems. In one study, supplementation with a cocktail diet containing cranberry showed greater richness in microbial diversity in the colonic lumen and colonic mucosa of piglets compared to the piglets fed on the antibiotic-containing diet. This diet was found to increase the presence of *Lactobacillus amylovorus* in both the colonic lumen and mucosa in piglets compared to those fed with the antibiotic or basal diets [62]. Furthermore, following the supplementation of a cocktail diet containing cranberry, the relative abundances of *Clostridiaceae, Peptostreptococcaceae* and the family *Streptococcaceae* were found to be reduced in weanling piglets, whereas the *Veillonellaceae* was increased. In addition, feeding weanling piglets with the cocktail diet containing cranberry plus colostrum was found to be associated with a reduction in the presence of the *Erysipelotrichaceae* family as well as a marked increase in the presence of the *Lactobacillaceae* family [63].

Dietary fibers are beneficial for the metabolic and physiological processes of the organism [64]. It was observed that feeding on a dietary fiber bundle that contained cranberry improved stool quality in dogs with chronic enteritis/gastroenteritis. Moreover, this fiber bundle caused a shift in gut bacteria from digesting mainly protein to digesting mainly carbohydrates. In addition, the fecal levels of several bioactive metabolites with beneficial antioxidant or anti-inflammatory properties increased in the dogs after the consumption of fiber-bundle-containing food [65]. In cats, feeding with a diet containing a 4% fiber bundle resulted in a decrease in levels of ammonium and fecal-branched-chain fatty acids (BCFAs). As high levels of ammonia and BCFAs cause putrefactive metabolism, their decrease after the use of a 4% fiber bundle resulted in a shift toward saccharolytic metabolism. In addition, feeding a fiber bundle to the cats resulted in increases in beneficial metabolites, like polyphenols hesperidin, hesperetin, ponciretin, secoisolariciresinol diglucoside, secoisolariciresinol, and enterodiol [66].

The beneficial effects of cranberry in improving gut microbiota were also studied in other animals and in humans. In one study, the exposure of mice to dextran sodium sulfate (DSS) caused a significant alteration in the fecal microbiota and a decrease in α diversity (diversity within a particular area or ecosystem). However, cranberry treatment not only improved the DSS-induced decrease in α-diversity but also reversed the alteration of the gut microbiota in colitic mice by enhancing the abundance of potentially beneficial bacteria, like *Lactobacillus* and *Bifidobacterium*, and reducing the abundance of potentially harmful bacteria, such as *Sutterella* and *Bilophila* [21]. In another study, oral supplementation with cranberry polyphenol was found to selectively and robustly increase the relative abundance of the metabolically beneficial bacterium *Akkermansia muciniphila* in mice. Other glycan-degrading bacteria, such as *Muribaculum intestinale, Bacteroides uniformis*, *Faecalibaculum rodentium*, and *Bacteroides acidifaciens,* were also stimulated by cranberry polyphenol mixed with a neo-fructan known as agavins. In this way, cranberry polyphenol not only maintains the gut microbiota composition but also regulates key mucosal markers that participate in the restoration of epithelial barrier integrity in mice [53]. The structural integrity of gut epithelial cells was also found to be maintained by cranberry fruits via an increase in tight junction genes such as occludin, tight junction protein 1 (TJP1), and mucin [67].

Cranberry and its constituents largely modulate the gut microbiome by decreasing the amount of pathogenic *Enterobacteriaceae* and enhancing the amount of the *Bacteroidaceae* gut bacterial family. In a gut simulator model, the cranberry component salicylate decreased the amount of *E. coli* and increased *Bacteroidaceae* [34]. In another gut simulator model, freeze-dried cranberry powder increased the abundance of luminal Bacteroidetes in the proximal colon of the simulator and decreased the abundance of Proteobacteria. Cranberry also markedly enhanced the levels of short-chain fatty acids (like acetate, butyrate, and propionate), while decreasing levels of branched-chain fatty acids [54]. Thus, cranberries acted as prebiotics with the utilization of host microorganisms and showed potential health-related effects relating to the suppression of pathogens and the selective stimulation of beneficial metabolites.

In addition to experimental models, cranberry was also found to be beneficial in shaping the human gut microbiota. In *Helicobacter pylori*-positive subjects, the supplementation of cranberry juice (480 or 240 mL) significantly reduced the abundance of Pseudomonas in the gut [55]. Supplementation with a cranberry extract capsule (two capsules per day providing 109.3 mg of polyphenols and 125 mg of oligosaccharides per day) for 4 days was shown to induce a strong bifidogenic effect in the human gut. The bifidogenic effect is a growth stimulation of *bifidobacteria*, which are a type of gut bacteria that metabolize a variety of complex carbohydrates. Cranberry extract also increased the abundance of several butyrate-producing bacteria, like *Clostridium* and *Anaerobutyricum*. Along with these effects, cranberry extract consumption has been shown to modify plasmatic and fecal short-chain fatty acid profiles with a reduction in the acetate ratio and an increase in the butyrate ratio [56]. Thus, the consumption of cranberry fruits can lead to significant improvements in gut health.

### 3.7. Bioactivity of Cranberry Polyphenols with Metal Conjugation

Cranberries contain a large number of polyphenols, including a variety of flavonoids. Although flavonoids have broad bioactivities related to health and diseases, conjugation with metals modulates metal homeostasis and plays an important role in their bioactivities. Using NMR and mass spectroscopy, it has been observed that flavonoids have zinc-binding sites. Zinc has been found to bind to the 3-hydroxyl-4-keto, catechol, and 5-hydroxyl-4-keto chelation sites of flavonol, 3′,4′-dihydroxylflavone, and chrysin, respectively. The binding of zinc induces distinct changes in the proton resonances on the flavonoid rings [68]. Besides zinc, cranberry polyphenols like quercetin, chrysin, 3-hydroxy flavone, 3′,4′-dihydroxy flavone, rutin, and flavone have strong iron-binding properties in aqueous media, as observed using UV/vis, NMR, and EPR spectroscopies and ESI-mass spectrometry. The “iron-binding motifs” have been identified in the structures of polyphenols that allow iron to bind strongly. Studies have shown that the binding of metal with plant polyphenols increases antioxidant and anti-inflammatory effects and enhances immune responses [69,70].

In view of this, metal nanoparticles were prepared using cranberry polyphenols. Ashour et al. [71] synthesized silver nanoparticles with cranberry powder aqueous extracts (0.2%, 0.5%, and 0.8% *w*/*v*). However, silver nanoparticles synthesized using a 0.2% extract exerted considerable in vivo wound healing efficacy in rats [71]. Another study showed that silver nanoparticles with cranberry juice have a broad spectrum of antimicrobial activity. Cranberry was found to be most active against pathogenic *bacteria Staphylococcus aureus, Bacillus subtilis*, and *B. cereus*, and was comparatively less active against fungus *Candida albicans* and foodborne *B. cereus* [72]. Recently, another palladium nanoparticle synthesized with cranberry fruit extract showed antibacterial activity by suppressing the growth of both Gram-positive and Gram-negative bacteria. It exhibited an anticancer effect on the MCF-7 breast cancer cell line [73]. The anti-inflammatory effect of the silver nanoparticles with cranberry polyphenols was also investigated in both in vitro (on HaCaT cell line, exposed to UVB radiation) and in vivo (on acute inflammation model in Wistar rats) settings. It was found that this combination has potent anti-inflammatory activity [74]. These studies indicate that metal nanoparticles synthesized with cranberry fruit extract have wound-healing, anti-inflammatory, and anticancer effects, as well as efficacy against large varieties of pathogenic bacteria.

The cranberry with zinc preparation (with vitamin C) showed effectiveness against paraoxonase activity. Paraoxonase 1 (PON1) is an enzyme that protects against vascular disease and organophosphate poisoning. It is also a biomarker for diseases that involve oxidative stress, inflammation, and liver disease. Supplementation of the cranberry extract (2 g/day) and vitamin C + Zn (300 mg/day) for 4 weeks showed an increase in the activity of paraoxonase 1 in nonsmoker healthy volunteers. The combination of cranberry and zinc also increased the total antioxidant status in the nonsmoker subgroup [75]. Thus, the cranberry and zinc combination have high antioxidant potential as well as paraoxonase 1 activity. The cranberry and zinc combination also helps patients suffering from periodontal diseases. It was observed that the addition of a multi-nutrient supplement, containing cranberry and zinc, showed a reduction in Stage III and IV periodontal disease when compared with a placebo [76]. Thus, cranberry with metals, particularly zinc, has beneficial effects on overall health improvement.

## 4. Cranberry in Animal Health

**Figure 3:**
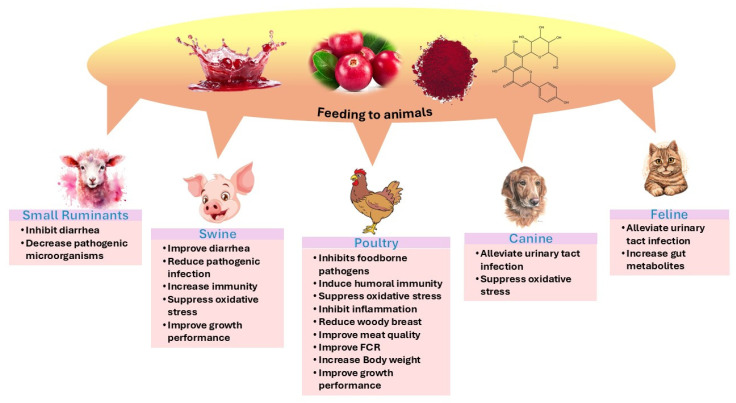
The effects of cranberry-based diet feeding on poultry, swine, canine, feline, and ruminant animals regarding their health and performance, including increases in daily weight gain by animals. FCR: feed conversion rate.

Cranberry has received considerable attention in animal health because it demonstrates potential pharmacological activities, like antioxidant [4], anti-inflammatory [13], antibacterial [14], antiviral [15], and immunomodulatory effects [20]. These inherent properties of cranberry may benefit animals by helping them to stay healthy, increasing the weight of farm animals, improving reproduction, and combating environmental stress (Figure 3) (derived from Table 2). Table 2 shows the beneficial effects of cranberry in livestock.

### 4.1. Cranberry in Poultry Health

Cranberries have multiple effects on the growth and development of poultry. It has efficacy, not only in boosting immunity, but also in fighting against various infectious diseases caused by poultry. For example, foodborne pathogens in the poultry industry, including bacteria, viruses, and other agents, are a major concern due to their association with foodborne illness and economic losses. Cranberry fruit has been found to be effective against various pathogens including Salmonella species, Campylobacter species, Listeria species, etc. In a study, an ethanolic extract of cranberry pomaces showed effectiveness against *Salmonella enterica* with minimum inhibitory (MIC) and bactericidal (MBC) doses of 8 and 16 mg/mL, respectively. Moreover, both anthocyanins and non-anthocyanin polyphenols of cranberry had lower MIC and bactericidal values of 4 mg/mL, indicating their higher antibacterial efficacy compared to cranberry pomace. Furthermore, it has been observed that cranberry polyphenol downregulates bacterial genes that are involved in flagellar motility, Salmonella Pathogenicity Island-1 (SPI-1), cell wall/membrane biogenesis, and gene transcription [77]. Another study showed that cranberry inhibits the production of *L. monocytogenes* by the competitive inhibitor of proline as cranberry polyphenols behave as proline analogs. Cranberry and oregano (1:1) with 2% sodium lactate inhibit the production of Listeria strains in both broth and cooked meat [78].

Cranberry pomace in poultry feed also has great influence on the chicken cecal microbiota and blood metabolites. Cranberry feed supplementation has been found to reduce *Eimeria acervulina* and *Clostridium perfringens* incidence in poultry. Cranberry treatments also impacted the population of *Lactobacillaceae*, *Enterobacteriaceae, Clostridiaceae*, and *Streptococcaceae* [79]. Because of this antimicrobial activity, cranberry-supplemented edible films were made from a mixture of whey proteins and chitosan. The edible films, placed on fresh turkeys, were shown to stop the microbiological deterioration of turkey meat and the development of pathogenic microorganisms *S. typhimurium*, *E. coli*, and *Campylobacter jejuni* for at least six days [80].

In a broiler model, NDMs showed a potent humoral immune response. In 7-day-old chicks vaccinated with the infectious bursal disease virus (IBDV) vaccine and fed with NDMs (0, 2, 4, or 8 mg/mL/bird orally) supplemented diet for 5 consecutive days, NDMs showed antioxidant activity and also comparatively five-fold higher than the cranberry juice. Interestingly, NDMs exerted anti-inflammatory activities comparable to Naproxen, but better than those of Ibuprofen. NDMs also increased the sensitivity of *S. aureus* to phagocytosis by chicken heterophils. In addition, NDMs showed increased serum IgM levels and their antibody titers against IBDV, suggesting that NDMs enhance bacterial vulnerability to immuno-defense mechanisms [52]. Another study also showed the efficacy of cranberry in the modulation of immunity in broiler chickens. Cranberry pomaces at 1% and 2% were found to increase IgY levels and upregulate the anti-inflammatory IL-10 gene in the blood serum of the birds, which indicates that feed supplemented with cranberry modulates innate immunity and inhibits the production of pro-inflammatory cytokines in chickens [79].

The meat quality of chicken is a rising issue in the poultry industry as the severity of woody breasts is increasing. However, cranberry pomace was found to be effective in improving meat quality. Cranberry pomace (0.5% in diet) was found to increase antioxidant capacity and decrease the severity of woody breasts in birds. The antioxidant capacity of cranberry pomace (0.5 or 1.0%) supplementation was associated with increased levels of antioxidant enzymes Nrf, Gpx2, and HO-1 [26]. Besides these applications, the use of cranberry in poultry production has been reported to have benefits on poultry performance. Cranberry pomace has been shown to increase the body weight of birds, particularly during the grower phase. Birds fed with cranberry pomace (0.5%) also showed a better feed conversion rate (FCR) compared to the same dose of blueberry pomace but had poorer FCR values than bacitracin methylene disalicylate. FCR measures the livestock’s production efficiency, which is the ratio of weight of feed intake to weight gained by the animal. Moreover, cranberry pomace (0.5%) showed the lowest mortality during the chicken’s grower phase compared to blueberry pomace [81].

### 4.2. Cranberry in Swine Health

Pigs can experience a variety of health issues, including infectious diseases at any stage of life, particularly after weaning. Postweaning diarrhea, and/or edema is, a common disease in piglets caused by enterotoxigenic or verotoxigenic *E. coli*, expressing F4 or F18 fimbriae. These fimbriae help in colonizing the bacteria in the intestinal mucosa and produce enterotoxins that cause diarrhea [92]. Cranberry extract (20 μg or 100 μg/mL) showed strong suppressing activity on F4+ *E. coli* and F18+ *E. coli* adherence. Cranberry extract (10 mg or 100 mg) also abolished the in vivo binding of F4 and F18 fimbriae to the pig intestinal epithelium in ligated loop experiments. Piglets that only received cranberry extract in feed (1 g/kg or 10 g/kg) did not show effects, but supplementation with feed (10 g/kg) and drinking water (1 g/L) significantly reduced excretion and diarrhea [82].

The use of weaning diets enriched with mixed feed additives (containing cranberry) can mitigate the effect of Salmonella infection on intestinal microbial populations and improve systemic and intestinal immune defenses. It was found that the use of mixed feed additives with colostrum in piglets increased the percentage of immune γδ T cells as well as the expression of the antioxidative *GPX2* gene. Piglets fed the diet of mixed feed additive with colostrum also exhibited a rapid change in ileal microbiota, with reductions in the *Erysipelotrichaceae, Streptococcaceae, Clostridiaceae*, and *Peptostreptococcaceae* family and increases in the *Lactobacillaceae* family [63]. This mixed feed additive, used alone or in combination with colostrum, increased the concentrations of vitamins E and B12 in the serum of piglets. In addition, this diet displayed the ability to improve the growth performance of piglets reared under commercial conditions [62]. After consumption, cranberries are metabolized and excrete different forms of oligosaccharide, which exert antiadhesive activities. In a study, adult female sows were fed with cranberry powder (5 g/kg/day) and it was found that collected urine fractions had antiadhesion activity when tested in a human red blood cell (A+) anti-hemagglutination assay with uropathogenic P-fimbriated *E. coli*. Later, it was found that sow urine contains no proanthocyanidins but a complex series of oligosaccharides, including arabinoxyloglucan. These arabinoxyloglucan oligosaccharides were structurally related to those found in cranberry, with antiadhesion properties found in urine after cranberry consumption [83].

Cranberry extract also has antimicrobial effects on pork slurry, pork burgers, and cooked ham. Cranberry pomace ethanol extract (2%) has shown significant growth suppression of pathogenic *L. monocytogenes* in both non-inoculated and inoculated bacteria pork slurry, pork burgers, and cooked ham [84]. Xi et al. [85] demonstrated that cranberry powder at 1%, 2%, and 3% showed 2–4 log cfu/g less growth of *L. monocytogenes* when compared to the control with nitrite alone [85]. In addition, the extract effectively suppressed the formation of lipid peroxidation indicator malondialdehyde in meat products, indicating its antioxidative properties [84]. Cranberry anthocyanin also inhibited the growth of *S. aureus* strains with MIC 5 mg/mL, although 2.0 MIC completely inhibited this pathogen in cooked pork and beef. Cranberry anthocyanin reduced intracellular ATP and soluble protein levels, damaged the membrane structure, and caused the leakage of cytoplasm, resulting in bacterial death [86]. Thus, cranberries exert effective antimicrobial effects and exhibit a natural preservative effect that suppressed the growth of food pathogens.

### 4.3. Cranberry in Canine Health

The UTI caused by *E. coli* is very common in humans and is also found in pets, including dogs. However, cranberry is found to be effective in suppressing uropathogenic *E. coli* growth in the urine of dogs fed with cranberry powder. Cranberry-fed female dogs’ but not male dogs’ urine had remarkable suppression effects in terms of bacterial adherence to MDCK cells compared to the animal-consuming control diet [88], indicating that cranberries offer protection to female dogs against the adhesion of uropathogenic *E. coli* to urinary epithelial cells. Another study also supported the notion that cranberry is effective in suppressing UTIs in dogs. Cranberry extract supplementation to the dogs for 6 months completely prevented the development of UTIs. Furthermore, bacterial adherence to MDCK cells was found to cause remarkable inhibition in the cranberry extract-fed dog’s urine samples, obtained at 30 and 60 days, compared to the urine samples obtained before extract feeding [40].

As a natural antioxidant, cranberry not only reduces oxidative stress in animals but also preserves dog food by preventing its oxidation during storage. The storage of dog food for 12 days at 55 °C after the addition of the cranberry 0.2% prevented the formation of thiobarbituric acid-reactive substances (TBARS) [93], indicating that cranberry prevented the oxidation of dog food.

### 4.4. Cranberry in Feline Health

Cats also develop UTIs from bacteria in their bladder or urethra, often causing the urethra to become obstructed, or preventing the proper emptying of the bladder. While urinating, cats manifest a symptom of pain or discomfort (dysuria), which can feel like burning, stinging, or itching, and also a clinical sign of a lower UTI (periurea). In a study, it has been found that a daily oral supplement of cranberry extract resulted in the reduction in lower urinary tract and gastrointestinal signs in feline idiopathic cystitis. On day 60 of treatment, cranberry extract supplementation caused the disappearance of lower urinary tract signs in cats [88].

Besides UTIs, chronic kidney disease can frequently occur in cats aged over 9 years. A nutraceutical diet containing cranberry powder was shown to manage chronic kidney disease in cats. In cats, 90 days of nutraceutical diet supplementation resulted in a decrease in creatinine, blood urea nitrogen, total proteins, and aspartate aminotransferase. A nutraceutical diet also decreased urine the turbidity score, color score, and the total proteins in cats. Cranberry-containing nutraceuticals were found to improve key indicators of renal failure in cats affected by chronic kidney disease [88]. Another study found that fiber bundle (where cranberry powder was a part) supplementation caused a change in fecal bacterial metabolism in cats. Fiber bundle consumption decreased levels of ammonium and fecal-branched-chain fatty acids (BCFAs) and increased the presence of beneficial metabolites from baseline values in cats [66].

### 4.5. Cranberry in Ruminant Health

Gastrointestinal nematodes are among the major health issues for small ruminants as they cause anemia, poor body condition, diarrhea, and death. It has been found that both aqueous cranberry and organic proanthocyanidin extracts are able to suppress *Haemonchus contortus* nematode larva and adult worm motility. The in vivo effect of aqueous cranberry and organic proanthocyanidin extracts was also observed in the small ruminant lamb model. Feeding 21.1 g cranberry powder to infected lambs for three consecutive days inhibited post-hatch L1 (EC_50_ 0.3 mg PAC/mL) and adults’ motility, as determined by collecting fecal matter [90], indicating cranberry and its polyphenols have utility in the integrated control of *H. contortus*.

The use of cranberry concentrate as a natural food preservative has also been evaluated in meat such as beef. As cranberry has displayed a potent antimicrobial effect, it has been observed that cranberry concentrate (2.5%, 5%, and 7.5%) reduced the total aerobic bacteria and *E. coli* O157:H7 when compared to the control [91]. These findings not only suggest that cranberry can be used as a natural preservative, but that it can also be used in controlling diarrhea and other infectious diseases.

## 5. Conclusions

Although cranberries are known for their high antioxidant content, they also exert anti-inflammatory, antimicrobial, and immunomodulatory properties. Cranberries are often associated with potential benefits for urinary tract health and digestive health due to the presence of polyphenolic compounds like anthocyanins, flavonoids, phenolic acids, tannins, and triterpenoids. Accumulated evidence showed that cranberries are effective in various forms, including their extract, powder, nondialyzable materials, and isolated polyphenols. Irrespective of its forms, cranberry induces the production of antioxidants by increasing antioxidant enzymes and suppressing lipid peroxidation, exhibits anti-inflammatory effects by suppressing inflammatory cytokines, exerts antimicrobial activity by suppressing bacterial adhesion and growth, and enhances immunity by modulating immune cells. Cranberry also helps in balancing gut microbiota by increasing the expression of probiotic bacteria and suppressing pathogenic bacteria in the intestines.

Cranberries have several health benefits for livestock, including poultry, swine, canine, feline, and ruminant animals, when used as either feed or supplements. Various studies revealed that cranberries are efficacious in controlling infectious diseases in poultry caused by Salmonella species and Campylobacter species. As postweaning diarrhea is a very common disease in piglets, caused by enterotoxigenic or verotoxigenic, cranberries are found to be beneficial in improving diarrheal symptoms and piglet mortality. Cranberry supplementation also decreases the signs of UTIs in dogs and cats by suppressing bacterial adherence. It also fights against nematodes in small ruminant animals and prevents infectious diseases. Because of this antimicrobial action, cranberry can reduce the use of antibiotics against infections. Regarding safety, the U. S. Pharmacopeial Convention concluded that the inclusion of cranberry ingredients is safe when consumed properly in dietary supplements and is not known to be associated with serious risks to health [94].

Along with the beneficial health effects of polyphenols, their harmful effects have also been reported in different experimental models [95]. Cranberry is considered to be generally well tolerated without adverse events. However, a high dose may result in minor side effects like diarrhea, abdominal discomfort, and nausea. Another possible side effect of cranberry juice or extracts could be seen in food–drug interactions, with reduced plasma levels and effects of proton pump inhibitors and histamine type 2 (H2) blockers [96]. However, it is believed that the right and recommended dose is safe and beneficial for health.

There is abundant evidence that cranberry has favorable effects on oxidative stress, inflammation, immunity, and infection. However, the currently available data mainly focus on in vitro and laboratory animal models. Some other studies on livestock, determining the beneficial effects of cranberries, are not very convincing at this stage due to lack of sufficient supportive and mechanistic data. As cranberry comprises various polyphenols, it is not clear which polyphenol is responsible for a specific activity. Therefore, more studies on livestock models are encouraged to provide enough evidence on beneficial effects of cranberry and its polyphenols.

## Figures and Tables

**Table 1:** Antioxidant, anti-inflammatory, antimicrobial, immunomodulatory, and gut-microbiota-balancing effects of cranberry.

Effect	Model	Dose	References
***Antioxidant Property***			
Inhibited ROS and induced CAT and SOD	*S. cerevisiae*	5–50 μg/mL	[22]
Exhibited antioxidant activity	DPPH, ABTS	6.44 & 12.30 mg TE/g	[23]
Exhibited antioxidant activity	ABTS, DPPH, and FRAP assays	0.04 gm/ml	[4]
Induced antioxidant effects	Hypercholesterolemic rats	5–10% *w*/*w* cranberry powder	[24]
Increased antioxidant status (SOD) and reduced MDA	Orchidectomized rats	27% and 45% cranberry juice	[25]
Increased levels of Nrf, Gpx2, and HO-1 antioxidant enzymes	Broilers	0.5 or 1.0% cranberry pomace	[26]
***Anti-inflammatory Property***			
Suppressed cytokine IL-8 and IL-6 and upregulated anti-inflammatory cytokine IL-10	LPS-stimulated macrophage	50 and 100 µg/mL cranberry concentrates	[13]
Inhibited pro-inflammatory cytokine IL-1β, IL-6, IL-8, and TNF-α	LPS-stimulated macrophage	cranberry juice concentrate	[27]
Neutralized virulency of *P. gingivalis* by inhibition of IL-8, CCL5 and NF-κB	Oral epithelial cells	100 μg/mL cranberry proanthocyanidins	[28]
Altered TNFα, IFIT1 and 3, MSR1, and CSF2 expression	THP-1 cells	100 μg/mL cranberry extract	[29]
***Antimicrobial Activity***			
Inhibited growth of wide range of human pathogenic bacteria	Gram +ve and gram −ve bacteria	50 µL of 5% of cranberries	[14]
Bactericidal effect of oral pathogens	*A. naeslundii and S. mutans*	0.50 mL/mL cranberry juice	[30]
Suppressed pathogenic bacteria	*S. aureus*	173.2 µg GAE/mL	[31]
Disrupted the biofilm formation and decreased swarming motility	*P. aeruginosa*	100 μg/mL cranberry PACs	[32]
Restricted virulence of *P. aeruginosa*	*Drosophila melanogaster*	200 μg/mL of cranberry extract	[33]
Reduced Enterobacteriaceae and Bacteroidaceae and protected from UTI	A human gut microbiota (simulator)	Cranberry powder containing 1 mg/mL Salicylate	[34]
Beneficial effects on uncomplicated UTI	Human patients	36 mg PAC	[35]
Prevented of recurrent UTI	Healthy, adult women	2 × 18.5 mg daily	[36]
Prevented recurrent uncomplicated UTI	Pre-menopausal adult women	18 mg cranberry PACs and 5 × 10^8^ CFU probiotic microorganisms	[37]
Prevented low UTI recurrence	Women with at least 4 episodes of cystitis	Combination of propolis and cranberry	[38]
Suppressed undesirable bacteria (*Synergistaceae* and *Desulfovibrio*, and *Fusobacteriaceae*)	Broiler chicken	1 or 2% of cranberry pomace	[39]
Suppressed UTI development	Dogs	Cranberry powder (1 g for <25 kg; 2 g for ≥25 kg)	[40]	
***Anti-viral Activity***			
Decreased viral load of MNV-1, FCV-F9, MS2(ssRNA) and phiX-174(ssDNA) bacteriophage	CRFK or RAW 264.7 cells	Cranberry juice with 0.30–1.20 mg/mL of PAC	[15]
Inhibited dengue virus and zika virus binding to the host–cell	Adult zebrafish, human Huh7.5 and A549 cell lines	25 to 2000 µg/mL	[41]
Inhibited replication cycle of Hazara virus through direct interaction	Vero cells	3.125–100 μg/mL cranberry extract	[42]
Targeted main protease (M^pro^) enzyme of SARS-CoV-2	in vitro FRET enzyme inhibitory assay	9.98 μM-cyanidin 3-O-galactoside 23.58 μg/mL-cranberry extract	[43]
Induced the aggregation of rotavirus and caused the destruction of rotavirus capsid protein VP6	Monkey kidney epithelial cells	Crude cranberry juice	[44]
Reduces rotavirus titer by 32%	Coliphage T4II (phage T4) and the rotavirus strain SA-11(RTV)	EGCG-30 µg/mL and cranberry proanthocyanidin-25 µg/ml	[45]
Inhibited influenza A and B viruses’ (IAV, IBV) attachment and entry into target cells	MDCK cells	2.5–20 μg/mL of Oximacro	[46]
Abolished the infectivity of HSV1 and 2 particles by targeting viral envelope glycoproteins gD and gB	African green monkey kidney cells	100 μg/mL of PACs-A	[47]
Inhibited influenza virus-induced hemagglutination, suppressed viral replication	MDCK cells	125 μg/mL NDM of cranberry	[48]
Inhibited neuraminidase enzymatic activity of influenza A and B strains	the MUNANA method	187–1200 μg/mL NDM	[49]
***Immunomodulatory Effect***			
Upregulated innate immune genes, enhanced host immune response	*C. elegans*	2 mg/mL of WCESP	[20]
Induced immune-implicated proteins and genes	Sprague Dawley rats	700 μg/rat/day	[50]
Enhanced human γδ-T immune cell proliferation	Human	450 mL of cranberry juice	[51]
Increased serum IgM level and their antibody titers against IBDV	Broilers	Cranberry NDM (2 and 4 mg/mL/bird)	[52]
***Gut Microbiota Enhancement***			
Increased beneficial bacteria like Lactobacillus and Bifidobacterium, and decreased harmful bacteria Sutterella and Bilophila	*DSS-induced colitis in mice*	1.5% (*w*/*w*) freeze-dried whole cranberry powder	[21]
Increased metabolically beneficial bacteria *Akkermansia muciniphila* and other glycan-degrading bacteria	*Obesogenic mice*	200 mg/kg	[53]
Increased short-chain fatty acids and decreased branched-chain fatty acids and increased luminal Bacteroidetes	*Human gut simulator*	5 g/L cranberry powder	[54]
Reduced abundance of *Pseudomonas*	*Humans*	480 mL cranberry beverage	[55]
Decreased acetate ratio and increased butyrate ratio by increasing butyrate-producing bacteria	*Healthy humans*	60 g of fresh cranberries	[56]
Increased beneficial bacterial taxa (*Bifidobacterium*, unclassified_*Rikenellaceae*, and *Faecalibacterium*) in intestine	Broilers	1% or 2% cranberry pomace	[39]

ROS—reactive oxygen species; CAT—catalase; SOD—superdioxide dismutase; WCESP—water-soluble cranberry extract standardized to 4.0% proanthocyanidins; DPPH—2,2-diphenyl-1-picrylhydrazyl); ABTS—2,2′-azino-bis(3-ethylbenzothiazoline-6-sulfonic acid); FRAP—ferric ion reducing antioxidant potential; LPS—lipopolysaccharide; IL—interleukin; TNF—tumor necrosis factor; NF-κB—nuclear factor–kappaB; MDA—malonaldehyde; CCL5—C-C motif chemokine ligand 5; CSF2—colony-stimulating factor 2; MSR1—macrophage scavenger receptor 1; IFIT—interferon-induced protein with tetratricopeptide repeats; GAE—gallic acid equivalent; UTI—urinary tract infection; PAC—proanthocyanidins; CFU—colony forming unit; MNV—murine norovirus; FCV—feline calicivirus; FRET—fluorescence resonance energy transfer; DSS—disodium sulfate.

**Table 2:** The effect of cranberry and its constituents on poultry, swine, feline, canine, and ruminant animals.

Type of Cranberry (e.g., Extract, Powder, etc.)	Dose and Route	Effects	Reference
***Poultry***			
Ethanolic extract from cranberry pomaces	2 or 4 mg/mL of cranberry pomaces	Downregulation of bacterial genes such as SPI-1 and hilA	[77]
Oregano and oregano–cranberry (1:1) ratio	750 ppm in both broth and cooked meat	Cranberry inhibits *L. monocytogenes*	[78]
Cranberry (CP1) and wild blueberry (BP1) pomace	1% each supplemented with food	Reduces *Eimeria acervulina* and *Clostridium perfringens* incidences and decreases intestinal lesion	[79]
Whey protein-chitosan film incorporating cranberry juice	1:1 ratio on fresh turkey	Inhibits multiple bacterial growth such as *S. typhimurium*, *E. coli*, and *C. jejuni*	[80]
NDMs of cranberry extract	1, 2, 4, 8 mg/mL oral	Increases humoral immune response and antioxidant, anti-inflammatory, and bacterial susceptibility to immuno-defense mechanisms	[52]
Cranberry pomace	0.5 or 1.0% diet	Increases levels of antioxidant enzymes Nrf2, Gpx2, and HO-1	[26]
Cranberry pomace	0.5% diet feed	Lowers mortality during the chicken’s grower phase	[81]
***Swine***			
Spray-dried cranberry powder	1% *w*/*w* in feed, 0.1% *w*/*v* in drinking water	Inhibited diarrhea by suppressing adherence of F4 and F18 fimbriae of *E. coli* to pig intestinal epithelium	[82]
Mixture of diets containing cranberry	1 g/kg cranberry and other polyphenol and vitamins in feed	Mitigates the influence of Salmonella infection on intestinal microbial populations and modulates systemic and intestinal immune defenses	[63]
Mixture of diet containing cranberry	0.1% cranberry and other polyphenol and vitamins in feed	Increases the concentrations of vitamins E and B12, improve the growth performance of piglets	[62]
Spray-dried cranberry powder	5 g/kg/day in diet to adult female sows	Arabinoxyloglucan oligosaccharides found in cranberry have antiadhesion properties.	[83]
Cranberry pomace ethanol extract	2% in pork slurry, pork burgers, and cooked ham	Significant growth inhibition of pathogenic *L. monocytogenes* and some other species	[84]
Cranberry powder	1%, 2%, and 3% in cured cooked pork	Inhibits 2–4 log CFU/g growth of *L. monocytogenes*	[85]
Cranberry anthocyanin	MIC 5 mg/mL in cooked pork and beef	Inhibits 8 log CFU/mL of S. aureus by lowering intracellular ATP and soluble protein levels and damaging membrane structure	[86]
***Canine***			
Cranberry fruit powder	2% of diet in dogs	Might provide protection to female dogs against adhesion of uropathogenic *E. coli* to urinary epithelial cells	[87]
Cranberry extract	1 g for dogs < 25 kg and 2 g for dogs ≥ 25 kg mixed with food	Prevents the development of a UTI and prevents E coli adherence to MDCK cells	[40]
***Feline***			
Oral nutritional supplement containing cranberry extract	60 mg cranberry extract per tablet with supplement	Reduces lower urinary tract and gastrointestinal signs in feline idiopathic cystitis.	[88]
Nutraceutical diet containing cranberry	0.0371% Cranberry	Decreases creatinine, blood urea nitrogen, total protein, aspartate aminotransferase, and urine turbidity score in cats	[89]
Fiber bundle composed of pecan shells, flax seed, and powders of cranberry, citrus, and beet	1%, 2%, and 4% in diet	Decreases levels of ammonium and fecal-branched-chain fatty acids and increased beneficial metabolites from baseline in cats.	[66]
***Ruminant***			
Cranberry vine powder	21.1 g for 3 days orally	Inhibits *Haemonchus contortus* in lambs	[90]
Cranberry concentrate	2.5%, 5%, and 7.5% (*w*/*w*) in beef	Reduces total aerobic pathogenic bacteria and *E. coli*	[91]

NDM—nondialyzable material; Nrf2—nuclear factor erythroid 2-related factor 2; GPx2—glutathione peroxidase 2; HO-1—heme oxygenase-1; MIC—minimum inhibitory concentration; CFU—colony forming unit; UTI—urinary tract infection.
